# Tératome sacro-coccygien: à propos d'un cas

**DOI:** 10.11604/pamj.2015.20.160.5553

**Published:** 2015-02-20

**Authors:** Moncef Chagou, Khadija Bernoussi

**Affiliations:** 1 Service de Gynécologie Obstétrique Cancérologie et Grossesse à haut risque, Maternité Souissi, Université Mohammed V, Rabat, Maroc

**Keywords:** Tératome sacro-coccygien, tumeur benigne, hydramnios, sacrococcygeal teratoma, benign tumor, hydramnios

## Image en medicine

Le tératome sacro-coccygien est une maladie rare, le plus souvent bénigne. Nous rapportons le cas d'une patiente avec une grossesse mal suivie et chez qui l’échographie lors d'un bilan étiologique d'hydramnios a objectivé un tératome sacro-coccygien type I qui a été pris en charge par les chirurgiens pédiatres après la naissance. Le pronostic de cette tumeur dépend de la précocité du diagnostic et traitement à laquelle il faut toujours penser en cas d'hydramnios associé. Le tératome sacro-coccygien est une maladie rare (1 nouveau-né sur 35 000-40 000) qui se présente le plus fréquemment chez les enfants de sexe féminin. Il s'agit d'une tumeur le plus souvent bénigne, détectable avant la naissance. Nous rapportons un cas de nouveau né présentant un tératome saccro-coccygien. La mère est une cinquième geste, cinquième part avec antécédent de trois césariennes, elle a été transférée dans notre maternité de niveau III pour exploration d'hydramnios sur une grossesse estimée à 34 semaines d'aménorrhées. A ce moment nous pensions essentiellement à trois diagnostics, un méningocèle myelomeningocèle et un tératome sacro-coccygien. Nous avons réalisé une échographie qui a objectivé un tératome sacro-coccygien type I sans signes d'anémie au doppler ou d'anasarque foetal. Il s'agit d'un processus lésionnel appendu au coccyx de contour lobulé d’écho-structure mixte à composante liquidienne majoritaire et composante tissulaire vascularisé au doppler avec grande citerne à 10cm. Une césarienne a été réalisée à 39 SA qui a permis l'extraction d'un nouveau né de sexe féminin avec un poids de naissance de 3200g et un Apgar 10/10 (A et B). Le nouveau né a été pris en charge à J2 de vie par une équipe de chirurgie pédiatrique.

**Figure 1:**
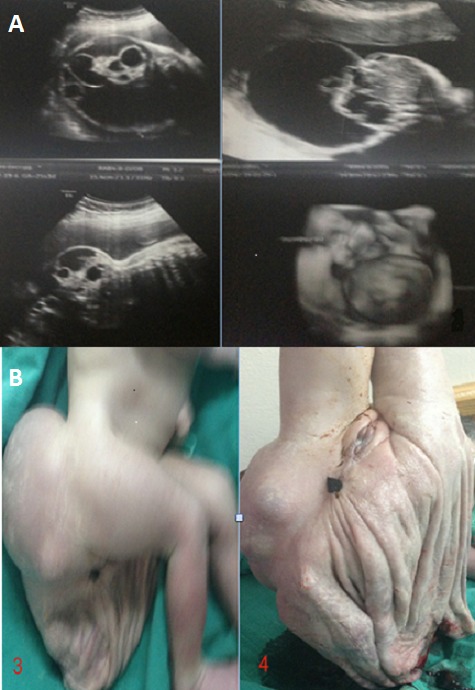
(A) échographie montrant un tératome sacro-coccygien type 1 à développement principalement externe avec composante pré sacrée minimal, le tout avec notion d'hydramnios manifeste; (B) tératome rompu après extraction

